# Path analysis of the awareness status and influencing factors of sarcopenia in older adults in the community: based on structural equation modeling

**DOI:** 10.3389/fpubh.2024.1391383

**Published:** 2024-07-24

**Authors:** Dahui Wang, Huaying Zeng, Peiwen Wu, Yuchen Zhou, Hongkun Chen, Falin Zhao, Shucong Liu

**Affiliations:** School of Public Health, Hangzhou Normal University, Hangzhou, China

**Keywords:** older adult, sarcopenia, awareness, influencing factors, pathway

## Abstract

**Background:**

Sarcopenia is a progressive geriatric syndrome that impacts older adults’ quality of life. Insufficient focus has been given to sarcopenia among Chinese residents, resulting in low level of sarcopenia awareness. This study aims to investigate awareness of sarcopenia and its influencing factors and the influencing pathways among older adults in Hangzhou.

**Methods:**

A stratified random sample of 942 community-dwelling older adults was evaluated using the SARC-CalF screening tool, along with a questionnaire based on health ecology theory to assess awareness of sarcopenia and its influencing factors and the influencing pathways. Descriptive statistics, linear regression analysis, and path analysis were conducted using SPSS 25.0 and Amos 23.0 to analyze the data.

**Results:**

The mean awareness score for sarcopenia was 60.26 ± 7.31. Self-rated physical health, daily intake of high-quality protein, exercise frequency, smoking status, self-efficacy, religious beliefs, social support, education level, occupation, participation in community free medical examinations, and awareness of nutrition policy were all factors affecting scores for sarcopenia awareness (*p* < 0.05). Except for negative effects observed in social support, smoking status, and self-rated physical health, all others exhibit positive effects.

**Conclusion:**

Community healthcare institutions should target populations with low awareness of sarcopenia and focus on these key factors. Diverse health education programs and multi-channel screening activities can promote awareness, guide healthy lifestyles and prevent or delay the onset of sarcopenia in the older.

## 1 Introduction

The global issue of population aging is currently a major concern. In China, the older adult population is growing rapidly, with an estimated 280 million individuals aged 60 and above by the end of 2022, accounting for 19.8% of the total population (1). As the aging process intensifies, there is growing attention towards sarcopenia, which is characterized by the progressive loss of skeletal muscle mass, accompanied by a decline in muscle strength and/or muscle function associated with age (2, 3). Sarcopenia is influenced by several important risk factors, including aging, malnutrition, physical inactivity, smoking, alcohol abuse, and the presence of chronic diseases (4). Available data indicates that the prevalence of sarcopenia among individuals aged 65 and above in China varies from 14 to 33%, with a further increase to 50 to 60% among those aged 80 and above (5).

Sarcopenia is a progressive geriatric syndrome that detrimentally impacts physical function and capability. It has an insidious onset and can result in functional impairments, thereby increasing the risk of falls, disability, hospital readmission, and mortality among older adults (5). Studies have demonstrated that muscle mass declines at a rate of 2.5 to 3.0% per year after the age of 60 (6). A 10% reduction in muscle mass can compromise immune function and heighten the susceptibility to infections, whereas a 20% decrease can lead to muscle weakness, limited capacity for daily activities, and an elevated risk of falls (7).

In China, approximately 40 million individuals suffer from severe injuries caused by sarcopenia-related falls each year (8). This condition greatly compromises both their quality of life and overall health. In addition, sarcopenia is intricately linked to and reciprocally influences several chronic conditions including diabetes, cardiovascular diseases, chronic obstructive pulmonary disease, osteoporosis, and cognitive impairment (9–11). Given that both sarcopenia and chronic diseases are widespread among older adults, they impose substantial economic and caregiving burdens on families and society (12). According to reports, the hospitalization expenses for sarcopenia patients surged to a staggering $40.4 billion in the United States in 2014 (13). This substantial financial burden underscores the urgent need to address sarcopenia as a critical public health concern in an aging society.

Hence, early prevention and management of sarcopenia are crucial, emphasizing preventive approaches, enhancing older adults’ awareness of sarcopenia, increasing their understanding of risk factors, and promoting preventive behaviors to mitigate the incidence and progression of sarcopenia from its roots (14). Studies have revealed inadequate attention towards sarcopenia among Chinese residents, with insufficient emphasis on its pathogenesis, risk factors, and potential harm. As a result, the overall awareness level regarding sarcopenia is low (15, 16). Disease awareness is influenced by various factors, including age, education level, occupation, and social environment. Thus, this study adopts the framework of ecological health theory, a significant theoretical model in public health practice. This theory highlights that individual health outcomes are the product of combined influences from genetic, psychological and behavioral, social and environmental, medical policy, cultural customs, and other factors (17).

This study conducted a comprehensive analysis on the factors and the pathways influencing the awareness of sarcopenia among older adults residing in the community. It evaluated the current status and identified existing issues regarding the awareness of sarcopenia in this population. The study also explored effective approaches to enhance the awareness of sarcopenia among older adults, presenting a foundation for conducting health education and research on sarcopenia in community-dwelling older adults.

This study puts forward the hypothesis that the current sarcopenia awareness among older adults is suboptimal. Furthermore, it postulates that disease awareness is influenced by various factors, including individual characteristics, psychological and behavioral lifestyles, family interpersonal networks, living and working environments, as well as policy and cultural contexts. And these influencing factors may interact with each other, collectively affecting older adults’ awareness of sarcopenia.

## 2 Materials and methods

### 2.1 Study design and sample

This study investigated the awareness of sarcopenia among Chinese older adults from October and November 2022. The sampling method in this study comprised two stages. Initially, survey sites were chosen through stratified random sampling. Subsequently, older participants were selected via convenience sampling at the designated survey sites. The selected areas encompassed Gongshu District, Xihu District, Yuhang District, and Linping District in the central urban area of Hangzhou city, Zhejiang Province, China. From each district, two streets were randomly selected to sample the older adults. The sample size was determined using the Kendall sample size rough estimation method, which recommends a sample size of 10–20 times the number of survey indicators (18). The questionnaire consisted of 41 items. Considering the response rate and efficiency, the sample size was augmented by 10%, resulting in a final sample size of 902 participants.

From October 2022 to November 2022, 942 older adults were surveyed using a stratified random sampling method in Hangzhou, Zhejiang Province, China. The study enrolled older adults residing in Hangzhou city as participants. The inclusion criteria were as follows: ① aged 60 years and above; ② permanent residents of the community for more than six months; ③ possessing clear consciousness, self-reporting no hearing impairments, cognitive and memory-related disorders, reading and expressive ability issues, or difficulties in communication with the researchers; and ④ voluntary participation with signing of an informed consent form. The exclusion criteria included meeting any of the following conditions: self-reported lack of autonomy or presence of end-stage diseases.

### 2.2 Ethics statement

This research protocol received approval from the Institutional Review Board of Hangzhou Normal University (Hangzhou, China, Approval No. 2022-1117) and was conducted in accordance with the Helsinki Declaration and ethical guidelines. Written informed consent was obtained from all respondents, ensuring the protection of privacy and confidentiality of personal information for the older adult participants.

### 2.3 Measurements

This study designed a questionnaire based on relevant literature to evaluate the awareness levels of sarcopenia in the older adults, along with related influencing factors and their pathways of impact.

#### 2.3.1 The questionnaire for assessing factors influencing awareness of sarcopenia

The questionnaire for assessing factors influencing awareness of sarcopenia in older adults was developed based on the health ecology theory. It consisted of five parts: (1) The personal characteristics considered in this study included gender, age, body mass index (BMI), waist circumference, presence of chronic diseases, self-rated health status, self-rated mental status, ability to perform daily living activities, and results of sarcopenia screening; (2) Psychological and behavioral lifestyle factors included smoking, alcohol consumption, exercise frequency, exercise duration, daily intake of high-quality protein, use of nutritional supplements, frequency of eating out, dietary regularity, and self-efficacy; (3) Family and interpersonal network factors included religious beliefs, marital status, living arrangement, and social support; (4) Living and working environment factors included occupation, education level, household monthly *per capita* income, availability of community life support services, and utilization of free community physical examination services; (5) Policy and cultural environmental factors included type of medical insurance, awareness of national nutrition policies such as the “Chinese Residents’ Dietary Guidelines,” and other relevant nutritional policies.

Sarcopenia screening in this study was conducted using the SARC-CalF (SARC-F combined with calf circumference) screening tool proposed by Barbosa-Silva et al. (19). A score of ≥11 points indicated a positive sarcopenia screening result. The SARC-CalF scale has demonstrated high sensitivity and specificity (20). Self-efficacy was assessed using the validated Chinese version of the General Self-Efficacy Scale, which has been widely utilized in the Chinese population. The scale demonstrated good reliability and validity, with a Cronbach’s alpha coefficient of 0.89 (21). The scale comprised 10 items, and the total score ranged from 10 to 40, with higher scores indicating greater levels of self-efficacy. Social support was assessed using the Social Support Rating Scale developed by Shuiyuan Xiao, and the scale demonstrated good reliability and validity, with a Cronbach’s alpha coefficient of 0.92 and a test-retest reliability coefficient of 0.89 (22). The Social Support Rating Scale consisted of a total score ranging from 12 to 66 points, with higher scores indicating greater levels of social support. Scores below 22 were classified as low, scores between 23 and 44 were moderate, and scores between 45 and 66 were high.

#### 2.3.2 The questionnaire for sarcopenia awareness assessment

The questionnaire for sarcopenia awareness assessment in older adults was designed based on the “Expert Consensus on Diagnosis and Treatment of Sarcopenia in Chinese Older Adults (2021) (4)“and the “Core Information Consensus on Preventing Sarcopenia in Chinese Older Adults (5).” The questionnaire underwent a pretest among 240 community older adults to assess its effectiveness and ensure the readability and clarity of its content. It consists of 15 items, and each item is rated on a 5-point Likert scale ranging from “completely disagree” to “completely agree.” Each item was scored on a 5-point Likert scale, with a maximum total score of 75. Higher scores were indicative of greater awareness. The awareness level was categorized as follows (23): a score ≥ 80% of the total possible score indicated good awareness level; a score ranging between 60 and 80% indicated moderate awareness level; and a score < 60% of the total possible score was considered poor awareness level. Awareness levels were classified based on the following criteria: scores of 60–75 were indicative of good awareness, scores of 45–59 indicated moderate awareness, and scores of 15–44 indicated poor awareness. The questionnaire was validated through investigation, with a Cronbach’s alpha coefficient of 0.841, KMO = 0.865, Bartlett *p* < 0.001, indicating good reliability.

### 2.4 Data collection

The researchers conducted one-on-one interviews for questionnaire collection. Prior to distributing the questionnaire, they provided a comprehensive explanation of the research purpose, process, benefits, and potential risks to all participating older adults. Only after ensuring that the older adults had a full understanding and willingly agreed to participate, they were asked to sign the informed consent form. For survey participants with a primary school education or below, the researchers provided assistance in completing the survey questionnaire. All participants who fill out the questionnaire will be rewarded with a small gift.

### 2.5 Statistical analysis

The database was established and data were entered using Epidata 3.1 software, while Statistical analysis was performed using SPSS 25.0 and AMOS 23.0 software. Professional verification was conducted to ensure data completeness and accuracy. Frequency and percentage were used to describe count data, while normally distributed measurement data were presented as mean ± standard deviation $\left(\bar{x}\pm s\right)$. Stratified linear regression analysis was employed to identify influencing factors. The structural equation model (SEM) was applied to analyze the pathways between various factors and sarcopenia awareness. All results were considered statistically significant at *p* < 0.05.

## 3 Results

### 3.1 Social and demographic characteristics

A total of 984 questionnaires were distributed, and 942 valid questionnaires were collected, resulting in a valid response rate of 95.73%. Out of 942 community-dwelling older adults, 332 (35.24%) were male and 610 (64.76%) were female. The age group with the largest representation was 70–79 years, comprising 403 individuals (42.78%), followed by 60–69 years, which included 385 individuals (40.87%). A total of 794 individuals (84.29%) were married. The educational level of the majority of older adults was primary school or below, accounting for 53.08% (*n* = 500), while 243 individuals (25.80%) had completed junior high school. Regarding household monthly *per capita* income, 57.54% of older adults had an income between 2000 and 4,999 yuan, while 22.40% had an income below 2000 yuan (Table 1).

**Table 1:** General demographic information of the participants (*n* = 942).

Variables		*n* (%)
Gender	Male	332 (35.24)
	Female	610 (64.76)
Age	60–69	385(40.87)
	70–79	403 (42.78)
	80–89	138 (14.65)
	≧90	16 (1.70)
Marital Status	Married	794 (84.29)
	Widowed	129 (13.69)
	Divorced	3 (0.32)
	Unmarried	16 (1.70)
Educational level	Primary School and Below	500 (53.08)
	Junior High School	243 (25.80)
	High School/Junior College	123 (13.06)
	College and Bachelor’s degree or above	76 (8.06)
Occupation type	Government employee, public sector worker	176 (18.68)
	Corporate/ Company personnel	232 (24.63)
	Service worker	32 (3.40)
	Farmer	306 (32.48)
	Laborer	141 (14.97)
	Self-employed individual	27 (2.87)
	Other	28 (2.97)
Religious belief or not	No	753 (79.94)
	Yes	189 (20.06)
Household monthly *per capita* income (RMB)	<2000	211 (22.40)
	2000–4,999	542 (57.54)
	5,000–6,999	126 (13.38)
	7,000–9,999	54 (5.73)
	>10,000	9 (0.95)
Type of medical insurance	Basic medical insurance for urban worker	484 (51.38)
	Basic medical insurance for urban and rural resident	412 (43.74)
	Public medical insurance	36 (3.82)
	Other	10 (1.06)
BMI (kg/m^2^)	<18.5	31 (3.29)
	18.5–23.9	446 (47.35)
	24–27.9	347 (36.84)
	≥28	118 (12.52)
Waist circumference	Normal	577 (61.25)
	Abnormal	365 (38.75)
Whether you have chronic diseases	No	297 (31.53)
	Yes	645 (68.47)
	No	297 (31.53)
Self-assessed health status in the last 3 months	Poor	52 (5.52)
	Moderate	111 (11.78)
	Good	779 (82.70)
Self-assessed mental status in the last 3 months	Poor	16 (1.70)
	Moderate	57 (6.05)
	Good	869 (92.25)
Exercise frequency	Hardly exercise	161 (17.09)
	1–2 times/week	31 (3.29)
	3–5 times/week	39 (4.14)
	6–7 times/week	711 (75.47)
Exercise time per exercise (Minutes)	0–29	215 (22.82)
	30–59	328 (34.82)
	≥60	399 (42.36)
Do you smoke	No	859 (91.19)
	Yes	83 (8.81)
Do you consume alcohol	No	769 (81.63)
	Yes	173 (18.37)
Meal Regularity in the last 3 months	Irregular	5 (0.53)
	general	15 (1.59)
	Regular	922 (97.88)
Whether you can reach the nearest fitness facility/gym in your neighborhood within 15 min on foot	No	65 (6.90)
	Yes	877 (93.10)
Whether you can reach the nearest healthcare facility by walking for 15 min	No	71 (7.54)
	Yes	871 (92.46)
Whether you have used the free medical examination services provided by the community hospital in the last year	No	67 (7.11)
	Yes	875 (92.89)
Level of awareness of nutrition policies	Unfamiliar	847 (89.92)
	Moderate	39 (4.14)
	Familiar	56 (5.94)
SARC-CalF Classification	Non- sarcopenia	841 (89.28)
	Sarcopenia	101 (10.72)

### 3.2 Awareness of sarcopenia in community-dwelling older adults

The mean score for sarcopenia awareness among community-dwelling older adults surveyed was 60.26 ± 7.31. Insufficient awareness of sarcopenia was identified in 51.27% of the older adult population (Table 2).

**Table 2:** The level of awareness of sarcopenia in community-dwelling older adults (*n* = 942).

Grades	Number (%)	Scores $\left(\bar{x}\pm s\right)$
Poor (15 ~ 44points)	2 (0.21)	43.0 ± 1.41
Moderate (45 ~ 59points)	481 (51.06)	54.20 ± 3.33
Good (60 ~ 75points)	459 (48.73)	66.70 ± 4.10
Total	942(100.00)	60.26±7.31

Data are presented as n (%) or mean ± standard deviation.

### 3.3 Factors associated with awareness of sarcopenia in community-dwelling older adults

A stratified linear regression analysis method was employed in this study to examine the factors influencing sarcopenia awareness among community-dwelling older adults. The model fitness coefficients indicated a high fit for all models. Each additional level of the related factor resulted in a significant increase in the explanatory power of the regression model. Ultimately, after incorporating all relevant factors into the model, a total of 36.2% of the variance could be explained. The results indicated that the final model included 12 variables that demonstrated statistical significance. These variables encompassed self-rated physical health status, daily intake of high-quality protein, exercise frequency, smoking behavior, self-efficacy, religious beliefs, social support, education level, occupation, household monthly *per capita* income, participation in community free medical examinations, and knowledge of nutrition policies (Table 3).

**Table 3:** Regression results of factors influencing awareness of sarcopenia in community-dwelling older adults.

Dimensions	Variables	Model 1	Model 2	Model 3	Model 4	Model 5
		β (*SE*)	β (*SE*)	β (*SE*)	β (*SE*)	β (*SE*)
Personal	SARC-CalF questionnaire screening scores (ref.:<11)					
characteristics	Scores≥11	−0.095** (0.764)	−0.075* (0.716)	−0.021 (0.672)	−0.031 (0.677)	−0.025 (0.662)
	Self-assessed health status (ref: poor)					
	Moderate health	0.113* (1.231)	0.094 (1.153)	0.155** (1.075)	0.174*** (1.058)	0.156** (1.035)
	Good health	0.053 (1.092)	0.019 (1.029)	0.126* (0.969)	0.149** (0.955)	0.125* (0.936)
Psychological	Daily intake of good quality protein		0.152*** (0.487)	0.156*** (0.454)	0.104*** (0.467)	0.088** (0.460)
and	Weekly frequency of exercise (ref: no exercise)					
behavioral	Exercise 6–7 times/week		0.159* (1.067)	0.118* (0.997)	0.150** (0.979)	0.141* (0.957)
lifestyle	Smoke (ref: No)					
	Yes		−0.084** (0.777)	−0.087** (0.721)	−0.085** (0.717)	−0.087** (0.704)
	Self-efficacy		0.272*** (0.040)	0.280*** (0.037)	0.256*** (0.038)	0.257*** (0.037)
Family	Religious belief (ref: No)					
interpersonal	Yes			0.200*** (0.533)	0.240*** (0.536)	0.224*** (0.527)
network	Social support			−0.265*** (0.030)	−0.263*** (0.031)	−0.245*** (0.030)
Living and	Educational level (ref: primary and below)					
working	Junior high school				0.091** (0.547)	0.083* (0.535)
environment	High school/ Junior college				0.072* (0.711)	0.050 (0.698)
	College and Bachelor’s Degree or above				0.124** (0.972)	0.118** (0.952)
	Occupation Type (ref: other)					
	Service worker				0.117** (1.601)	0.097* (1.579)
	Household monthly *per capita* income (ref: < 2000 RMB)					
	2000–4,999 RMB				0.088* (0.537)	0.083* (0.525)
	Have you used free medical examination services provided by community hospitals in the last 1 year (ref: No)					
	Yes				0.079** (0.800)	0.070* (0.783)
Policy and	Level of awareness of nutrition policy (ref: Unfamiliar)					
cultural	Moderate awareness of nutrition policy					0.100*** (1.022)
environment	Familiar Nutrition Policy					0.167*** (0.869)
Regression	R2	0.029	0.163	0.283	0.331	0.362
model	ΔR2	0.029	0.134	0.119	0.048	0.032
	F	5.614	13.950	24.321	15.008	16.144
	*p*	<0.001	<0.001	<0.001	<0.001	<0.001

Stratified linear regression was performed with the awareness score of sarcopenia as the dependent variable. The regression model included the following stratified models: Model 1 Personal characteristics, Model 2 Psychological and behavioral lifestyle, Model 3 Family interpersonal network, Model 4 Living and working environment, and Model 5 Policy and cultural environment. For categorical variables, the “No” group was selected as the reference category. *P* < 0.05 represents statistical significance. *p < 0.05; **p < 0.01; ***p < 0.001.

### 3.4 Pathway analysis of awareness influencing factors of sarcopenia in community older adults

**Figure 1:**
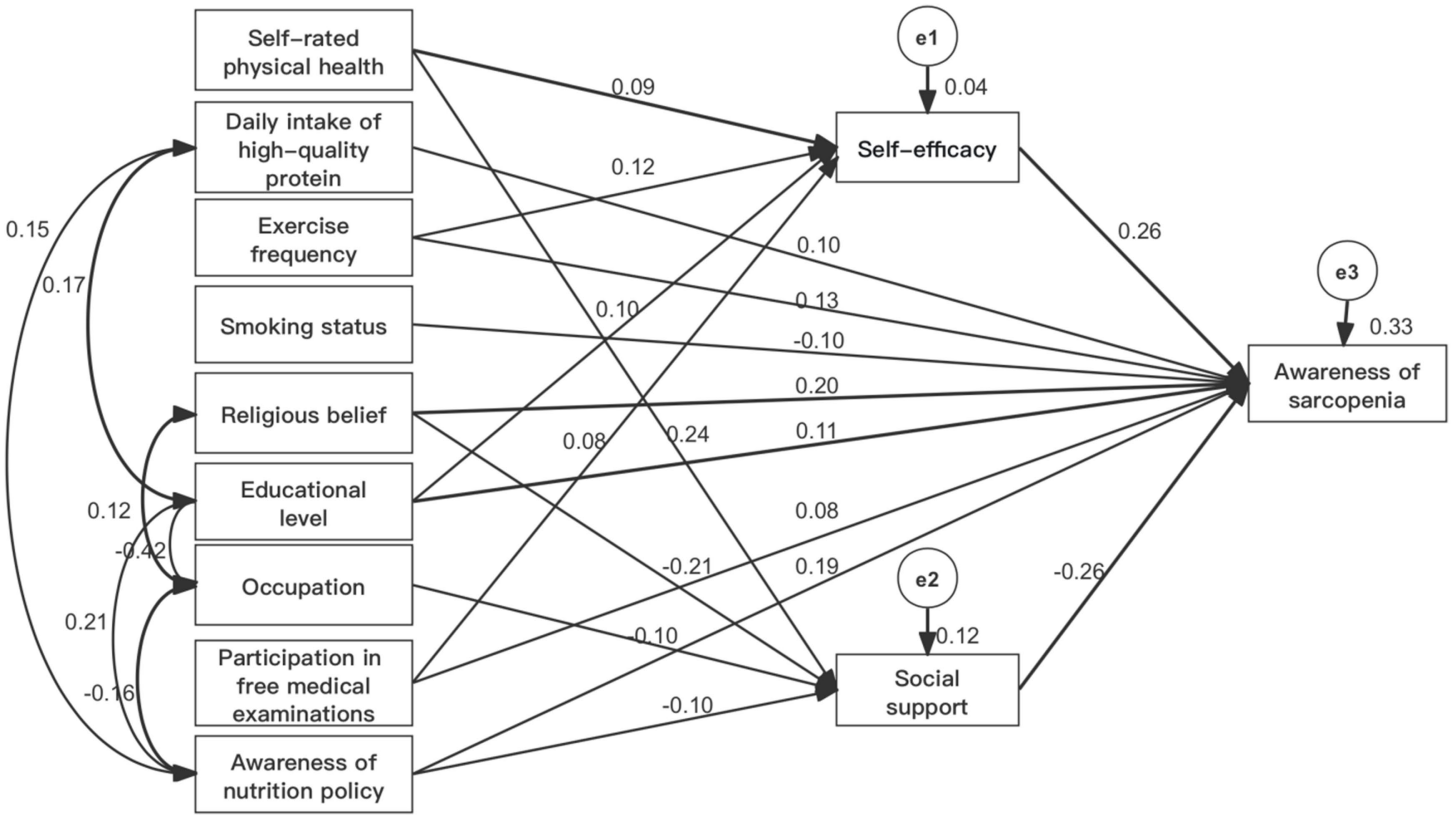
Standard path coefficients of the model.

Based on the results of linear regression analysis and relevant professional knowledge, a structural equation model of awareness influencing factors of sarcopenia was constructed. The research data were validated using the maximum likelihood method (ML). After the modification and deletion of non-significant paths, the model fit well (Table 4). The standardized path coefficients of the modified structural equation model for the awareness influencing factors of sarcopenia in older adults are shown in Figure 1. The effects of each variable on the awareness of sarcopenia in older adults are divided into direct effects, indirect effects, and total effects. The order of the total effects of each variable on the awareness of sarcopenia is as follows: self-efficacy (0.262) > social support (−0.260) > religious beliefs (0.255) > understanding of nutritional policies (0.219) > exercise frequency (0.164) > level of education (0.135) > smoking status (−0.099) > daily intake of high-quality protein (0.096) > use of free medical check-up services at the community hospital in the past year (0.095) > self-rated health status in the past 3 months (−0.037) > occupation (0.027). Except for social support, smoking status, and self-rated health status in the past 3 months, which have negative effects, the rest have positive effects (Table 5).

**Table 4:** The revised model fit analysis.

Indices	*χ*2/df	RMSEA	GFI	AGFI	CFI	IFI	NFI	TLI
Model fit value	3.063	0.047	0.977	0.958	0.906	0.909	0.870	0.856
Evaluation criterion	<5	<0.08	>0.90	>0.90	>0.90	>0.90	>0.85*	>0.85*

*>0.85 acceptable and >0.90 goodness. RMSEA = root mean square error of approximation; GFI = goodness of fit index; AGFI = adjusted goodness of fit index; CFI = -comparative fit index; IFI = incremental fit index; NFI = normed fit index; TLI = Tucker-Lewis index; df = degree of freedom.

**Table 5:** Summary of the impact effects of various factors on awareness of sarcopenia.

Pathways	Direct effect	Indirect effect	Total effect
Self-rated health status in the past 3 months → Awareness	-	−0.037	−0.037
Daily high-quality protein intake ratio → Awareness	0.096	-	0.096
Exercise frequency → Awareness	0.132	0.031	0.164
Smoking → Awareness	−0.099	-	−0.099
Religious beliefs → Awareness	0.201	0.054	0.255
Educational level → Awareness	0.108	0.027	0.135
Occupation → Awareness	-	0.027	0.027
Free community hospital medical check-up services in the past year → Awareness	0.075	0.020	0.095
Level of understanding of nutrition policies → Awareness	0.194	0.025	0.219
Self-efficacy → Awareness	0.262	-	0.262
Social support → Awareness	−0.260	-	−0.260

The Bootstrap method was used to test the mediating effects, and the results indicated that self-efficacy and social support partially mediated the relationship between self-rated health status in the past 3 months, exercise frequency, religious beliefs, level of education, occupation, use of free community medical check-up services in the past year, understanding of nutritional policies, and awareness of sarcopenia. The 95% confidence intervals of the mediating effects did not include 0 (Table 6).

**Table 6:** Results of the mediation analysis.

Pathways	Coefficient	95%CI		p
		Lower	Upper	
Self-rated health status in the past 3 months → self-efficacy/social support → Awareness	−0.037	−0.065	−0.01	0.007
Exercise frequency → self-efficacy → Awareness	0.031	0.016	0.048	0.001
Religious beliefs → social support → Awareness	0.054	0.037	0.074	0.001
Educational level → self-efficacy → Awareness	0.027	0.011	0.046	0.001
Occupation → social support → Awareness	0.027	0.01	0.045	0.001
Free community hospital health check-up service in the past year → self-efficacy → Awareness	0.020	0.007	0.032	0.005
Understanding of nutritional policies → social support → Awareness	0.025	0.01	0.042	0.003

## 4 Discussion

Among the surveyed participants, 48.73% demonstrated a good level of awareness regarding sarcopenia. A study by Soon Lean Keng et al. (24) on Malaysian residents aged 18 and above revealed that only 6.9% of the participants had a comprehensive understanding of the characteristics, consequences, and treatment of sarcopenia. Jeanine M. Van Ancum (25) discovered that merely 9% of Dutch residents were familiar with the concept of sarcopenia. Shu-Chun Lee (26) conducted a survey on older individuals aged 65 and above in Taipei, China, and found that their average score for sarcopenia awareness was 2.83 ± 0.82 (out of 5 score), indicating a limited level of familiarity with sarcopenia. At present, there is a scarcity of research regarding sarcopenia awareness, and the existing studies are in their preliminary stages. Researchers have utilized individually tailored assessment tools to conduct surveys, leading to discrepancies in both the tools employed and the demographics surveyed. Consequently, this has led to inconsistent research findings among different researchers.

However, a significant proportion (51.27%) of the older adult population exhibited insufficient awareness of sarcopenia, indicating the need for enhanced awareness, particularly considering its recent recognition as a disease by the World Health Organization (WHO) in 2016 (27). Sarcopenia is a geriatric disease with high prevalence and challenges associated with diagnosis (28). Currently, the social awareness of this disease remains relatively low. Based on reports, it was found that only 0.19% of the 1,056 nurses surveyed from 19 provinces in China possessed adequate knowledge of sarcopenia. Additionally, a significant majority (65.72%) of the nurses scored below passing grades in their assessment of sarcopenia knowledge (23). The inadequate level of disease awareness among healthcare professionals can impede the diagnosis and treatment of sarcopenia in clinical practice (29). Sarcopenia is characterized as a progressive disorder involving loss of muscle strength, muscle mass, or physical function (2, 3). However, these symptoms are often misconstrued as natural signs of aging. Additionally, in China, the absence of specialized geriatric departments in most hospitals has resulted in limited and inaccurate dissemination of knowledge concerning geriatric syndromes (23). While healthcare institutions place significant focus on preventing falls, their efforts primarily revolve around improving the physical environment and providing health education on medications that may induce falls. Consequently, sarcopenia, as an independent risk factor for falls tends to be overlooked (30). Therefore, it is crucial to increase public awareness of sarcopenia as an essential strategy for the effective prevention and treatment of both sarcopenia and its associated conditions (26).

In this study, Self-rated physical health has indirect effects on sarcopenia awareness through self-efficacy (0.024) and social support (−0.062). Older adults with good self-rated health tend to possess a stronger sense of control over their lives (31), strong sense of self-efficacy, enabling them to engage more actively with the outside world, acquire information, and consequently demonstrate higher levels of sarcopenia awareness. However, when individuals rate their own health as good, it reduces the motivation to seek medical and social support, weakening the potential informational function of social support, leading to decreased awareness of sarcopenia.

Older adults who maintain a healthy lifestyle and exhibit good psychological well-being demonstrate higher levels of sarcopenia awareness. The study results revealed that older adults who consumed high-quality protein on a daily basis, engaged in physical exercise 6–7 times per week, refrained from smoking, and demonstrated high self-efficacy exhibited higher scores of sarcopenia awareness. The effects of exercise frequency and daily high-quality protein intake on sarcopenia awareness are both positive (0.164, 0.096), while smoking status has a negative effect on sarcopenia (−0.099). This observation may be due to the greater emphasis placed by this group of older adults on their own health, as well as their heightened awareness of health-related issues and favorable daily habits (32). Prior research has demonstrated a positive correlation between disease knowledge and healthy lifestyle behaviors. Individuals possessing greater disease knowledge are more inclined to adopt healthy lifestyle behaviors, which can help prevent disease progression and associated complications (33). Previous research has identified a positive correlation between protein intake and protein knowledge (34). In our study, older adults with higher scores of sarcopenia awareness have a greater understanding of protein nutrition knowledge and a relatively higher protein intake.

In this survey, it was found that 8.81% of the older population were smokers, and their awareness of sarcopenia was lower compared to non-smokers, which is in line with previous studies (24). Non-smokers exhibited higher levels of health literacy compared to smokers (35), and the lower health literacy observed in smokers was associated with their insufficient knowledge regarding health-related matters (36). This study revealed a lower level of sarcopenia awareness among smokers, potentially attributed to their limited access to education and health information, inadequate awareness of the hazards associated with smoking, and a lack of active involvement in acquiring and mastering health-related knowledge and skills (37).

The study found that self-efficacy has the greatest impact on sarcopenia awareness (0.262), and it is a positive influence. Self-efficacy plays a crucial role in influencing individuals’ adoption of healthy lifestyles. Research has indicated that older adults with high self-efficacy are more confident in sustaining a healthy lifestyle and forming healthy habits (38). Additionally, there is a notable correlation between high self-efficacy and disease knowledge (39). Community healthcare institutions should prioritize the physical and mental well-being of the older adult and focus on fostering their self-efficacy. This approach can effectively enhance the impact of sarcopenia health education and facilitate older adults in modifying their unhealthy habits at an earlier stage.

We found that older individuals who hold religious beliefs exhibit higher scores of sarcopenia awareness. The influence of religious beliefs on sarcopenia awareness is positive (0.255). This may be attributed to their heightened social engagement compared to their non-religious counterparts (40). The problem-solving mechanisms and social support provided by their religious beliefs influence their daily behaviors and lifestyle, leading to enhanced mental well-being (41). Different religious beliefs advocate diverse dietary culture, inadequate protein intake is a significant risk factor for triggering sarcopenia. Therefore, people who choose vegetarianism because of their religious beliefs should consume sufficient additional plant-based protein to maintain muscle health (42). We also investigated the relationship between social support and sarcopenia awareness among older adults. In contrast to previous studies, this study found that older adults with low levels of social support had higher scores of sarcopenia awareness. The impact of social support on sarcopenia awareness is negative (−0.260). In traditional Chinese culture, the family holds a central role in social support (43), A strong family support system can expand the range of health-related information available to older adults (44) and provide them with enhanced healthcare resources (45). Nevertheless, research on sarcopenia in China is still at an early stage, with limited social awareness and public attention. Reliance solely on family support is inadequate for older adults to acquire sufficient knowledge about sarcopenia. Conversely, older individuals with lower levels of family support tend to rely more on medical services, presenting opportunities to obtain knowledge about sarcopenia from healthcare professionals during the treatment of associated chronic conditions. These findings highlight the importance for community healthcare institutions to not only foster self-health awareness among the older adults but also enhance the dissemination and accessibility of knowledge about sarcopenia. This can be achieved by raising public awareness and encouraging widespread attention to sarcopenia, while also leveraging the positive impact of the family support system to promote the health of older adults affected by sarcopenia.

The living and working environment has a significant impact on the awareness of sarcopenia in older adults. There is a positive correlation between their education level and scores of sarcopenic awareness. The effect of educational level on sarcopenia awareness is positive (0.135). Consistent with previous studies, individuals with higher levels of education possess a more comprehensive understanding of the disease (46), Furthermore, education is positively associated with the motivation to acquire knowledge, enhance awareness, and adopt healthier lifestyle choices (47). According to the Development Report on the Quality of Life for the older adults in China (2019) (48), compiled by the China Scientific Research Center on Aging, approximately 29.6% of older individuals in China have not received formal education, while approximately 41.5% have attained primary school education. The educational level of the older adults in China is generally low, therefore, we need to strengthen health education for the older adults even more. In this study, the occupation of older adults was found to influence their awareness of sarcopenia. The effect of occupation on sarcopenia awareness is positive (0.027). Specifically, older adults working in the catering service industry displayed the highest scores of sarcopenia awareness, potentially attributed to their occupation exposing them to a greater amount of nutrition-related information. This results in a positive impact on individuals’ awareness of sarcopenia.

This study revealed that older adults who utilized community-based free physical examination services in the previous year exhibited higher scores of sarcopenia awareness compared to those who did not utilize such services. Whether the use of community free medical check-up services has a positive effect on sarcopenia awareness (0.095). As of 2015, a total of 118 million older adults aged 65 and above in China were benefiting from the provision of free medical examination services (49). This could be attributed to their heightened concern for personal health status, proactive engagement with community doctors in case of abnormal situations, and expression of health needs, leading to a greater acquisition of health knowledge. These findings imply the need for community healthcare institutions to enhance awareness among older adults regarding the significance of health checkups, promote active participation in such checkups, and advocate for the inclusion of sarcopenia screening as part of routine examinations. These measures aim to enhance older adults’ awareness of and attention to sarcopenia.

Studies have revealed a positive correlation between the level of nutrition policy understanding among older adults and their scores of sarcopenia awareness. The overall effect of the level of understanding of nutrition policies on sarcopenia awareness is 0.219. This may be because older adults who pay more attention to policies have a better understanding of the risks and impacts of sarcopenia. As a result, they become more conscientious about their daily behaviors and lifestyle choices, aiming to prevent the onset of sarcopenia. These findings emphasize the importance of healthcare institutions reinforcing the promotion and dissemination of national nutrition-related policies, such as dietary guidelines for residents. By enhancing older adults’ self-care awareness, improving their knowledge regarding sarcopenia, and enabling accurate assessments of their own health, these efforts can potentially delay the onset and progression of sarcopenia.

The level of sarcopenia awareness among older individuals in the community in Hangzhou city is inadequate. Given the critical role of community settings in identifying and preventing sarcopenia, early screening and intervention (2, 3) are imperative to mitigate adverse health outcomes (50). Health education serves as a fundamental approach for community-based chronic disease management, playing a crucial role in enhancing health knowledge, fostering attitude change, improving skills, and facilitating the modification of unhealthy behaviors (51). Community healthcare institutions should focus on factors that affect sarcopenia awareness among older adults and implement specific health education and interventions. This includes actively promoting core information about sarcopenia prevention, guiding older adults to identify risk factors early (11), and encouraging healthy lifestyle habits to reduce negative health outcomes and alleviate the burden on healthcare resources.

## 5 Limitations

This study had certain limitations in sample selection, as it only focused on older individuals from specific areas in Hangzhou. To address this, future research can conduct comprehensive surveys across multiple regions and centers. Additionally, some variables in this study were evaluated based on self-reported data provided by the participants. Furthermore, being a cross-sectional study, this research cannot establish a causal relationship between healthy lifestyles and sarcopenia awareness in older adults. Therefore, further longitudinal studies are necessary to explore and understand these relationships in greater depth.

## 6 Conclusion

The current sarcopenia awareness among older individuals in the community in Hangzhou city requires improvement. The cultivation of sarcopenia awareness among older adults is intricately linked to their individual psychological and behavioral lifestyle, family interpersonal networks, as well as their living and working environments, along with policy environments. To address this issue, it is recommended to implement community-based diversified health education programs and multi-channel screening activities in the future. These endeavors will effectively enhance sarcopenia awareness among older adults in the community, guiding them towards the adoption of healthy lifestyle habits and the prevention and delay of sarcopenia occurrence.

## Ethics statement

The studies involving humans were approved by the Institutional Review Board of Hangzhou Normal University (Hangzhou, China, Approval No. 2022-1117). The studies were conducted in accordance with the local legislation and institutional requirements. The participants provided their written informed consent to participate in this study.

## Author contributions

DW: Conceptualization, Funding acquisition, Resources, Supervision, Writing – review & editing. HZ: Data curation, Investigation, Software, Writing – original draft. PW: Data curation, Investigation, Writing – original draft. YZ: Investigation, Software, Writing – original draft. HC: Investigation, Writing – original draft. FZ: Formal Analysis, Methodology, Writing – review & editing. SL: Conceptualization, Funding acquisition, Project administration, Writing – original draft.
