# Supplementary material for: Path analysis of the awareness status and influencing factors of sarcopenia in older adults in the community: based on structural equation modeling
Source: Front Public Health. 2024 Jul 24;12:1391383. doi: 10.3389/fpubh.2024.1391383 (PMC11304347; doi:10.3389/fpubh.2024.1391383)
Supplement: Supplementary file 1 [file Table_1.DOCX]

**Questionnaire on the Awareness and Influencing Factors of Sarcopenia among Older Adults in the Community**

Greetings! We are currently conducting a survey and sincerely invite you to participate. Please be assured that all of your responses will be kept strictly confidential and used solely for academic research purposes. We hope you will answer the following questions truthfully. Completing this questionnaire is expected to take 5-10 minutes. Thank you for your time and support. Please mark "✓" on the item you believe is most accurate.

**I. Demographic Information**

1. Gender: ① Male ② Female

2. Height: ______ cm

3. Weight: ______ kg

4. Waist Circumference: ①<85cm ②85-89cm ③90-99cm ④≥100cm

5. Age: ①60-69years ②70-79years ③80-89years ④≥90years

6.Ethnicity: ①Han Chinese ②Other

7. Religious Belief:

①None ②Yes (Buddhism, Islam, Catholicism, Christianity, Other)

8. Marital Status: ①Married ②Widowed ③Divorced ④Other

9. Living Arrangement: ①Living alone ②Not living alone

10.Educational Level: ①Elementary school or less ②Junior high school

③High school/Technical secondary school ④Junior college and above

11. Current or Former Occupation: ①Government or public sector worker

②Corporate/Company employee ③Service personnel ④Farmer

⑤Worker ⑥Self-employed ⑦Other

12. Is your current or former occupation related to medical or food nutrition fields?

①Yes ②No

1. Average monthly household income per capita: ①<2,000RMB ②2,000-4,999RMB ③5,000-6,999RMB ④7,000-9,999RMB ⑤≥10,000RMB

14. Type of medical insurance:

①Urban employee medical insurance ②Urban and rural residents medical insurance

③Publicly funded medical care ④Other

15.Please specify the daily intake of high-quality protein-rich foods:

①Eggs: ______ pieces

②Dairy products: ______ ml

③Meat, fish, and shrimp: ______ g

④Tofu and its products: ______ g

1. Do you take additional protein powder or other nutritional supplements?

①Yes (Type of supplements taken: multivitamins, calcium tablets, protein powder, fish oil, other)

②No

1. Weekly exercise routine, if you engage in multiple activities, please specify all (include type of exercise and duration):

①Rarely exercise or sedentary due to illness or bedridden

②1-2 times/week: Type of exercise ______; Duration per session ______

③3-5 times/week: Type of exercise ______; Duration per session ______

④6-7 times/week: Type of exercise ______; Duration per session ______

1. Do you smoke (if yes, please specify daily amount and years of smoking)?

①Yes Amount per day: ______ cigarettes/day; Smoking duration: ______ years

②No

19.Do you consume alcohol (if yes, please specify amount, frequency, and type of alcohol)?

①Yes Amount per occasion: ______ ml;

Drinking frequency: ①1-2 times/week ②3-4 times/week ③5-7 times/week ④>7 times/week;

Type of alcohol: ①Spirits ②Wine ③Yellow wine ④Beer ⑤Other

②No

20.Do you have any chronic diseases (if yes, please specify type and duration of illness)?

①Yes Type of illness (Diabetes, Hypertension, Obesity, Fatty liver, Gout, Hyperlipidemia, Coronary heart disease, Stroke, Cancer, Osteoporosis, Gastritis, Peptic ulcer, Other);

Duration of illness: ______ years (specify the longest duration)

②No

21. Regularity of diet in the past three months:

①Irregular ②Moderately regular ③Regular

1. Frequency of choosing takeout or dining out per week:

①<1 time ②1-2 times ③3-4 times ④5 or more times

23. Self-assessment of health status over the past three months:

①Poor ②Average ③Good

24. Self-assessment of mental state over the past three months:

①Poor ②Average ③Good

25. Self-assessment of daily living skills:

①Poor ②Fairly poor ③Good

1. Can you reach the nearest supermarket/vegetable market/fruit shop within a 15-minute walk from your community?

①Yes ②No

27. Can you reach the nearest fitness facility/gym within a 15-minute walk from your community?

①Yes ②No

28. Can you reach the nearest medical institution within a 15-minute walk?

①Yes ②No

29. Does your community provide free dining halls or free milk for the elderly?

①Yes ②No or unsure

30. Have you used any free health check-up services provided by the community hospital in the past year?

①Yes ②No

1. Are you familiar with the "Dietary Guidelines for Chinese Residents" and policies related to nutrition improvement and nutritional risk screening for the elderly outlined in the "14th Five-Year Plan for Healthy Aging"?

①Not familiar ②Moderately familiar ③Familiar

**II. SARC-CalF Questionnaire**

1.Muscle Strength: Difficulty in lifting/carrying an object weighing about 10 pounds (4.5 kg)

①No difficulty

②Some difficulty

③Considerable difficulty, unable to complete

2.Assisted Walking: Difficulty walking across a room

①No difficulty

②Some difficulty

③Considerable difficulty, requires assistance or unable to complete

3.Chair Stand: Difficulty standing up from a bed or chair

①No difficulty

②Some difficulty

③Considerable difficulty, cannot do it without help

4.Climbing Stairs: Difficulty climbing 10 steps

①No difficulty

②Some difficulty

③Considerable difficulty, unable to complete

5.Number of Falls: Number of falls in the past year

①0 times

②1 to 3 times

③4 times or more

6.Calf Circumference: Measure the circumference of the dominant calf with a 20 cm gap between the feet and legs relaxed

①Male >34 cm, Female >33 cm ②Male ≤34 cm, Female ≤33 cm

**III. Questionnaire on the Sarcopenia Awareness of Community Older Adults (Please select the option that best reflects your actual situation).**

| **Item** | **Strongly Disagree** | **Disagree** | **Neutral** | **Agree** | **Strongly Agree** |
| --- | --- | --- | --- | --- | --- |
| **1.**Sarcopenia is a geriatric syndrome, the prevalence of which increases with age. | ① | ② | ③ | ④ | ⑤ |
| **2.**Patients with sarcopenia may exhibit symptoms such as weakness, frequent falls, difficulty walking, slow gait, and frail limbs. | ① | ② | ③ | ④ | ⑤ |
| **3.**Older adults who take multiple medications are more susceptible to sarcopenia. | ① | ② | ③ | ④ | ⑤ |
| **4.**Prolonged bed rest, complete inactivity, sitting for long periods, and reduced physical activity can cause or exacerbate sarcopenia. | ① | ② | ③ | ④ | ⑤ |
| **5.**Older adults with inadequate intake of energy and protein are more likely to develop sarcopenia. | ① | ② | ③ | ④ | ⑤ |
| **6.**A weight loss of more than 5% within six months should prompt medical consultation, along with screening and risk assessment for sarcopenia. | ① | ② | ③ | ④ | ⑤ |
| **7.**Older adults, especially those experiencing frequent falls, should be screened for sarcopenia and assessed for associated risks. | ① | ② | ③ | ④ | ⑤ |
| **8.**I believe that appropriate dietary nutrition can help delay muscle deterioration in the elderly. | ① | ② | ③ | ④ | ⑤ |
| **9.**I consider appropriate dietary nutrition crucial for the prevention and management of common chronic diseases. | ① | ② | ③ | ④ | ⑤ |
| **10.**I believe that a reasonable diet can improve overall health status. | ① | ② | ③ | ③ | ⑤ |
| **11.**I am aware of the importance of actively participating in outdoor activities to enhance muscle health. | ① | ② | ④ | ④ | ⑤ |
| **12.**I am aware of the importance of actively participating in outdoor activities to enhance muscle health. | ① | ② | ③ | ③ | ⑤ |
| **13.**I consume various types of vegetables and fruits daily to maintain my health. | ① | ② | ③ | ④ | ⑤ |
| **14.**I consume a sufficient amount of high-quality protein daily, including fish, meat, eggs, dairy products, and soy. | ① | ② | ③ | ④ | ⑤ |
| **15.**I believe that regular health check-ups and nutritional assessments are beneficial for correcting unhealthy dietary habits and improving health status. | ① | ② | ③ | ④ | ⑤ |

IV. Self-Efficacy (Regarding your usual perception of yourself, please select based on your actual situation or feelings).

| Items | Strongly Disagree | Disagree | Agree | Strongly Agree |
| --- | --- | --- | --- | --- |
| **1.**If I try hard enough, I can always solve problems. | ① | ② | ③ | ④ |
| **2.**Even when others disagree, I can find a way to get what I want. | ① | ② | ③ | ④ |
| **3.**For me, sticking to my ideals and achieving my goals is effortless. | ① | ② | ③ | ④ |
| **4.**I am confident in my ability to handle unexpected situations effectively. | ① | ② | ③ | ④ |
| **5.**With my intelligence, I can manage unexpected situations well. | ① | ② | ③ | ④ |
| **6.**If I put in the necessary effort, I can solve most problems. | ① | ② | ③ | ④ |
| **7.**I can stay calm in the face of difficulties because I trust in my problem-solving abilities. | ① | ② | ③ | ④ |
| **8.**When faced with a problem, I can usually think of several solutions. | ① | ② | ③ | ④ |
| **9.**When I'm in trouble, I can usually think of something to do. | ① | ② | ③ | ④ |
| **10.**No matter what happens, I can handle it with ease. | ① | ② | ③ | ④ |

**V. Assessment of Social Support**

1.How many close friends do you have who can offer you support and assistance?

①None ②1-2 ③3-5 ④6 or more

2.In the past year, have you:

①Lived far from family and alone

②Frequently moved and lived mostly with strangers

③Lived with classmates, colleagues, or friends

④Lived with family

3.Your relationship with neighbors:

①No concern for each other, merely acquaintances

②Slight concern in times of difficulty

③Some neighbors are very caring

④Most neighbors are very caring

4.Your relationship with friends:

①No concern for each other, merely acquaintances

②Slight concern in times of difficulty

③Some colleagues are very caring

④Most colleagues are very caring

5.Support and Care Received from Family Members (Please mark '✓' in the appropriate box)

| (1) Spouse (Partner) | ①None | ②Very little | ③Moderate | ④Full support |
| --- | --- | --- | --- | --- |
| (2) Parents | ①None | ②Very little | ③Moderate | ④Full support |
| (3) Children | ①None | ②Very little | ③Moderate | ④Full support |
| (4) Siblings | ①None | ②Very little | ③Moderate | ④Full support |
| (5) Other family members (e.g., sister-in-law) | ①None | ②Very little | ③Moderate | ④Full support |

6.In the past, during emergency situations, sources from which you received financial support and practical problem-solving assistance included:

(1) No sources

(2) The following sources (multiple selections allowed):

①Spouse ②Other family members ③Relatives ④Friends

⑤Colleagues ⑥Workplace

⑦Political parties, trade unions, and other official or semi-official organizations

⑧Religious and social groups, and other non-official organizations

⑨Other

7.In the past, during emergency situations, sources from which you received comfort and concern included:

(1) No sources

(2) The following sources (multiple selections allowed):

①Spouse ②Other family members ③Relatives ④Friends

⑤Colleagues ⑥Workplace

⑦Political parties, trade unions, and other official or semi-official organizations

⑧Religious and social groups, and other non-official organizations

⑨Other

8.Your method of expressing distress:

①Never share with anyone

②Share only with 1-2 very close individuals

③Will discuss if friends ask about it

④Proactively share distress to gain support and understanding

9.Your approach to seeking help when facing troubles:

①Rely solely on myself, do not accept help from others

②Rarely ask for help from others

③Sometimes ask for help from others

④Often seek assistance from family, friends, or organizations when in difficulty

10.Your participation in group activities (e.g., political party, religious groups, senior associations):

①Never participate ②Occasionally participate ③Frequently participate

④Actively and enthusiastically participate

Investigator:

Date of Survey:

Community Surveyed:
