# Supplementary material for: Path analysis of the awareness status and influencing factors of sarcopenia in older adults in the community: based on structural equation modeling
Source: Front Public Health. 2024 Jul 24;12:1391383. doi: 10.3389/fpubh.2024.1391383 (PMC11304347; doi:10.3389/fpubh.2024.1391383)
Supplement: Supplementary file 2 [file Data_Sheet_1.PDF]

The questionnaire for sarcopenia awareness, consisting of 15 items, was subjected to the Kaiser-Meyer-Olkin (KMO) measure of sampling adequacy and Bartlett's test of sphericity. The results showed a KMO value of 0.865 and the Bartlett's test reached a significant level ($P < 0.001$), indicating the suitability of the data for factor analysis. Utilizing principal component analysis and maximum variance orthogonal rotation method, four common factors were extracted based on eigenvalues greater than 1, with a cumulative variance contribution rate of 62.063%. The scree plot indicated that the slope of the trend flattened after the fourth common factor, suggesting that the first four common factors explained a substantial amount of variance, while the contribution of the fifth common factor and beyond to the total variance was minimal (refer to Figure 1 for details). Consequently, it was determined to extract four common factors, and all items entered into the expected common factors, as detailed in Table 1.

Table 1. Factors with Eigenvalues > 1 and Explained Variables Extracted from the Second Exploratory Factor Analysis

Component	Initial Eigenvalues			Rotation Sums of Squared Loadings		
	Total	% of Variance	Cumulative %	Total	% of Variance	Cumulative %
1	5.18	34.535	34.535	3.889	25.926	25.926
2	1.517	10.116	44.651	2.247	14.978	40.905
3	1.452	9.682	54.333	1.607	10.71	51.615
4	1.160	7.731	62.063	1.567	10.449	62.063

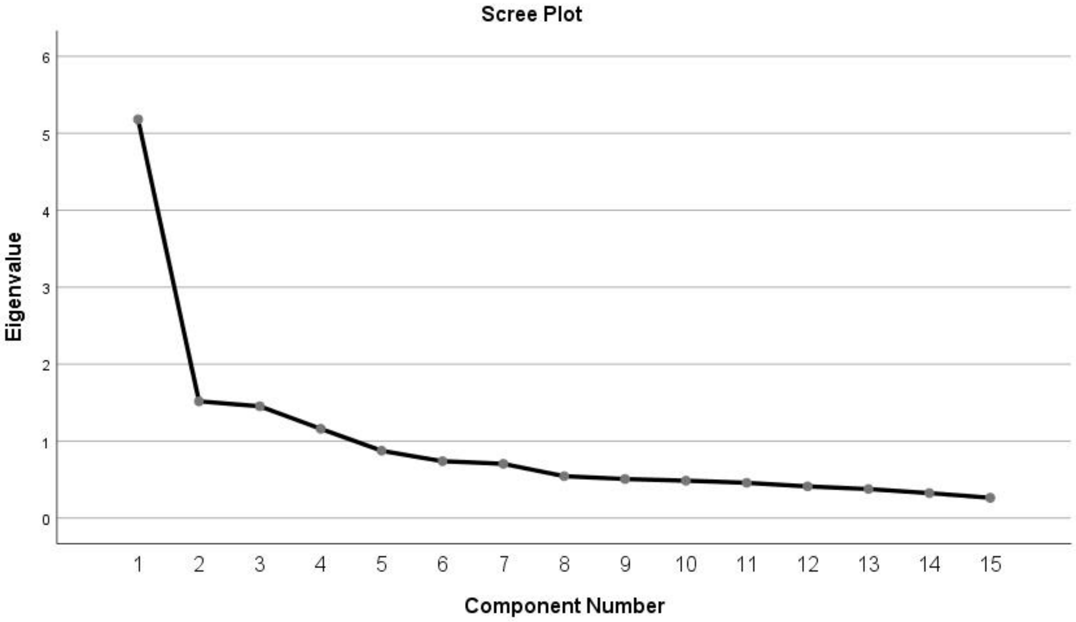

Figure 1. Scree Plot of the Second Exploratory Factor Analysis

In this study, Cronbach's α coefficient and split-half reliability tests were used to evaluate the overall reliability of the sarcopenia disease cognition scale. Cronbach's α coefficient is a method for determining reliability through internal consistency; the higher the α coefficient, the better the internal consistency of the questionnaire. Split-half reliability also evaluates internal consistency by dividing the items in the scale into two parts and calculating the correlation coefficient between them. An internal consistency reliability coefficient ≥0.8 for the entire scale and reliability indices ≥0.70 for subscales indicate good internal consistency of the evaluation questionnaire. The results showed that the Cronbach's α coefficient for the total evaluation scale was 0.841, and the split-half reliability for the total evaluation scale was 0.713, as detailed in Table 2.

Table 2 Results of Internal Consistency and Split-Half Reliability of the Assessment Scale

Indicators	Number of Items	Cronbach’s α Coefficient	Split-Half Reliability
Overall Assessment Scale	15	0.841	0.713
