# Supplementary material for: Path analysis of the awareness status and influencing factors of sarcopenia in older adults in the community: based on structural equation modeling
Source: Front Public Health. 2024 Jul 24;12:1391383. doi: 10.3389/fpubh.2024.1391383 (PMC11304347; doi:10.3389/fpubh.2024.1391383)
Supplement: Supplementary file 3 [file Data_Sheet_2.PDF]

The Univariate Analysis of Sarcopenia Awareness in Community-dwelling Older Adults

Univariate analyses were conducted to examine the awareness of sarcopenia among older adults living in the community. The analysis considered factors related to individual traits, psychological and behavioral lifestyle, family and community networks, living and working environment, as well as the policy environment. The results are shown in Table 1. At the individual trait level, significant differences were observed in the scores of sarcopenia awareness among older adults based on variables such as ethnicity, SARC-CalF questionnaire screening results, self-assessed mental health status, self-assessed physical health status, and daily activity ability ($P<0.05$). Regarding the psychological and behavioral lifestyle level, notable variations were found in the scores of sarcopenia awareness among older adults concerning key factors such as the adherence to dietary guidelines for daily high-quality protein intake, exercise habits, duration of each exercise session, smoking status, and self-efficacy ($P<0.05$). Regarding the family and community network, significant differences were observed in the scores of sarcopenia awareness among older adults based on variables such as religious beliefs and levels of social support ($P<0.05$). At the level of living and working environment, statistically significant differences in the scores of sarcopenia awareness among older adults were found pertaining to variables such as educational level, occupation, monthly family income, accessibility to the nearest fitness facility within a 15-minute walk in their community, and utilization of the community's free medical examination services ($P<0.05$). In terms of the policy and cultural environment, statistically significant differences were observed in the scores of sarcopenia awareness among older adults in relation to variables such as the type of medical insurance and the level of comprehension of nutrition policies ($P<0.05$).

Table 1 Univariate analysis of sarcopenia awareness (n=942)

**Table 1** Univariate analysis of sarcopenia awareness (n=942)			
**Variables**	**Numbers (%)**	**Scores ($\bar{x}\pm s$)**	***P***
Personal characteristics dimension			
Gender			
Male	332 (35.24)	60.17±7.18	0.774
Female	610 (64.76)	60.31±7.39	
Age			
60-69	385 (40.87)	60.52±7.21	0.757
70-79	403 (42.78)	60.07±7.34	
80-89	138 (14.65)	60.28±7.51	
≧90	16 (1.70)	59.06±7.88	
BMI（kg/m^2）			
＜18.5	31 (3.29)	59.16±7.60	0.109
18.5-23.9	446 (47.35)	60.81±7.09	
24-27.9	347 (36.84)	60.03±7.32	

**Table 1** Univariate analysis of sarcopenia awareness (n=942)			
**Variables**	**Numbers (%)**	**Scores ($\bar{x} \pm s$)**	***P***
⩾28	118 (12.52)	59.19±7.93	
Waist circumference (Waist circumference <85cm for women and <90cm for men is considered normal, and the opposite is considered abnormal.)			
Normal	577 (61.25)	60.63±7.21	0.052
Abnormal	365 (38.75)	59.68±7.44	
Whether you have chronic diseases			
No	297 (31.53)	60.65±7.56	0.273
Yes	645 (68.47)	60.09±7.20	
SARC-CalF Classification			
Non- sarcopenia	841 (89.28)	60.51±7.42	0.001[a]
Sarcopenia	101 (10.72)	58.24±6.01	
Self-assessed health status in the last 3 months			
Poor	52 (5.52)	59.54±6.73	0.006
Moderate	111 (11.78)	62.32±7.10	
Good	779 (82.70)	60.02±7.34	
Self-assessed mental status in the last 3 months			
Poor	16 (1.70)	62.75±6.82	0.001
Moderate	57 (6.05)	63.40±7.01	
Good	869 (92.25)	60.01±7.29	
Psychological and behavioral lifestyle dimension			
Exercise frequency			
Hardly exercise	161 (17.09)	58.06±7.52	<0.001[b]
1-2 times/week	31 (3.29)	56.19±5.89	
3-5 times/week	39 (4.14)	59.54±6.35	
6-7 times/week	711 (75.47)	60.98±7.22	
Exercise time per exercise (Minutes)			
0-29	215 (22.82)	58.67±7.47	0.001
30-59	328 (34.82)	61.13±7.34	
⩾60	399 (42.36)	60.41±7.08	
Do you smoke			
No	859 (91.19)	60.46±7.20	0.008
Yes	83 (8.81)	58.23±8.17	
Do you consume alcohol			
No	769 (81.63)	60.30±7.18	0.773[a]
Yes	173 (18.37)	60.11±7.92	
Meal Regularity in the Last 3 Months			
Irregular	5 (0.53)	55.60±11.87	0.082
general	15 (1.59)	57.07±6.20	
Regular	922 (97.88)	60.34±7.29	
Self-efficacy			<0.001

**Table 1** Univariate analysis of sarcopenia awareness (n=942)			
**Variables**	**Numbers (%)**	**Scores ($\bar{x} \pm s$)**	***P***
Daily intake of good quality protein			＜0.001
Family and interpersonal network dimension			
Religious belief or not			
No	753 (79.94)	59.39±7.10	＜0.001
Yes	189 (20.06)	63.74±7.13	
Marital status			
Married	794 (84.29)	60.25±7.23	0.102[b]
Widowed	129 (13.69)	60.7±7.83	
Divorced	3 (0.32)	65.67±4.04	
Unmarried	16 (1.70)	56.25±6.67	
Social support			＜0.001
Living and working environment dimension			
Educational Level			
Primary School and Below	500（53.08）	59.22±7.43	＜0.001
Junior High School	243（25.80）	61.2±7.09	
High School/Junior College	123（13.06）	61.29±7.12	
College and Bachelor's degree or above	76（8.06）	62.49±6.50	
Occupation type			
Government employee, public sector worker	176 (18.68)	60.93±6.52	0.001[b]
Corporate/ Company personnel	232 (24.63)	60.64±6.95	
Service worker	32 (3.40)	62.53±7.44	
Farmer	306 (32.48)	59.08±7.64	
Laborer	141 (14.97)	61.19±7.96	
Self-employed individual	27 (2.87)	61.78±6.05	
Other	28 (2.97)	57.18±6.73	
Household monthly per capita income (RMB)			
<2000	211 (22.40)	58.64±7.79	0.002
2000-4999	542 (57.54)	60.49±7.24	
5000-6999	126 (13.38)	61.16±6.90	
7000-9999	54 (5.73)	62.09±6.12	
>10000	9 (0.95)	61.33±7.35	
Whether you can reach the nearest fitness facility/gym in your neighborhood within 15min on foot			
No	65 (6.90)	62.08±6.68	0.038
Yes	877 (93.10)	60.13±7.34	

Table 1 Univariate analysis of sarcopenia awareness (n=942)			
**Variables**	**Numbers (%)**	**Scores ($\bar{x} \pm s$)**	***P***
Whether you can reach the nearest healthcare facility by walking for 15 min			
No	71 (7.54)	59.45±7.43	0.33
Yes	871 (92.46)	60.33±7.31	
Whether you have used the free medical examination services provided by the community hospital in the last year			
No	67 (7.11)	57.64±6.02	<0.001[a]
Yes	875 (92.89)	60.47±7.37	
Policy and cultural environment dimension			
Type of medical insurance			
Basic medical insurance for urban worker	484 (51.38)	60.60±6.97	<0.001[b]
Basic medical insurance for urban and rural resident	412 (43.74)	59.35±7.54	
Public medical insurance	36 (3.82)	65.78±7.33	
Other	10 (1.06)	62.10±4.10	
Level of awareness of nutrition policies			
Unfamiliar	847 (89.92)	59.60±7.08	<0.001[b]
Moderate	39 (4.14)	64.00±7.67	
Familiar	56 (5.94)	67.66±5.56	

Note: Data are presented as n (%) or mean ± standard deviation. For two independent samples that fit a normal distribution but have unequal variances, a corrected t-test is used, represented by [a]; when three or more independent samples are involved, the Kruskal-Wallis H-test is used as a multiple sample rank test, represented by [b]. Abbreviations: BMI, Body Mass Index.
