# Supplementary material for: Path analysis of the awareness status and influencing factors of sarcopenia in older adults in the community: based on structural equation modeling
Source: Front Public Health. 2024 Jul 24;12:1391383. doi: 10.3389/fpubh.2024.1391383 (PMC11304347; doi:10.3389/fpubh.2024.1391383)
Supplement: Supplementary file 4 [file Data_Sheet_3.PDF]

We categorized the level of sarcopenia awareness into three levels: good, moderate, and poor, and conducted single-factor and logistic regression analysis on the results.

Table 1 Awareness Level Grading and Univariate Analysis of sarcopenia

	Classification of Awareness Scores for Sarcopenia [n (%)]				
Variables	Poor（n=2）	Moderate（n=481）	Good（n=459）	x^2/F	P
Gender				x^2=0.565	0.926
Male	1（0.50）	171（35.60）	160（34.90）		
Female	1（0.50）	310（64.40）	299（65.10）		
BMI（kg/m^2）				x^2=8.293	0.200
＜18.5	0（0.00）	20（4.20）	11（2.40）		
18.5-23.9	1（0.50）	212（44.10）	233（50.80）		
24-27.9	1（0.50）	181（37.60）	165（35.90）		
≥28	0（0.00）	68（14.10）	50（10.90）		
Waist circumference (Waist circumference <85cm for women and <90cm for men is considered normal, and the opposite is considered abnormal.)				x^2=2.761	0.245
Normal	0（0.00）	297（61.70）	280（61.00）		
Abnormal	2（100.00）	184（38.30）	179（39.00）		
Age				x^2=2.799	0.970
60-69	1（0.50）	200（41.60）	184（40.10）		
70-79	1（0.50）	204（42.40）	198（43.10）		
80-89	0（0.00）	68（14.10）	70（15.30）		
≧90	0（0.00）	9（1.90）	7（1.50）		
Ethnicity				x^2=5.366	0.241
Han	2（100.00）	481（100.00）	457（99.60）		
Other	0（0.00）	0（0.00）	2（0.40）		
Religious belief or not				x^2=64.882	＜0.001
No	2（100.00）	433（90.00）	318（69.30）		
Yes	0（0.00）	48（10.00）	141（30.70）		
Marital status				x^2=9.772	0.309
Married	2（100.00）	404（84.00）	388（84.50）		
Widowed	0（0.00）	66（13.70）	63（13.70）		
Divorced	0（0.00）	0（0.00）	3（0.70）		
Other	0（0.00）	11（2.30）	5（1.10）		
Live alone or not				x^2=4.060	0.109
No	2（100.00）	432（89.80）	393（85.60）		
Yes	0（0.00）	49（10.20）	66（14.40）		
Educational Level				x^2=13.865	0.015
Primary School and Below	0（0.00）	277（57.60）	223（48.60）		
Junior High School	1（50.00）	114（23.70）	128（27.90）		
High School/Junior College	1（50.00）	60（12.50）	62（13.50）		
College and Bachelor's degree or above	0（0.00）	30（6.20）	46（10.00）		
Occupation type				x^2=25.893	0.004
Government employee, public sector worker	0（0.00）	79（16.40）	97（21.10）		
Corporate/ Company	1（50.00）	128（26.60）	103（22.40）		

personnel					
Service worker	0（0.00）	12（2.50）	20（4.40）		
Farmer	0（0.00）	167（34.70）	139（30.30）		
Laborer	1（50.00）	65（13.50）	75（16.30）		
Self-employed individual	0（0.00）	9（1.90）	18（3.90）		
Other	0（0.00）	21（4.40）	7（1.50）		
Household monthly per capita income (RMB)				x^2=15.243	0.056
<2000	0（0.00）	127（26.40）	84（18.30）		
2000-4999	2（100.00）	263（54.70）	277（60.30）		
5000-6999	0（0.00）	65（13.50）	61（13.30）		
7000-9999	0（0.00）	21（4.40）	33（7.20）		
≥10000	0（0.00）	5（1.00）	4（0.90）		
Type of medical insurance				x^2=19.541	0.003
Basic medical insurance for urban worker	2（100.00）	249（51.80）	233（50.80）		
Basic medical insurance for urban and rural resident	0（0.00）	221（45.90）	191（41.60）		
Public medical insurance	0（0.00）	8（1.70）	28（6.10）		
Other	0（0.00）	3（0.60）	7（1.50）		
The proportion of daily high-quality protein intake to the recommended dietary intake（ref: ≥100%）				x^2=15.913	0.004
<75%	1（50.00）	368（76.50）	308（67.10）		
75%-100%	1（50.00）	113（23.50）	149（32.50）		
≥100%	0（0.00）	0（0.00）	2（0.40）		
Whether supplementation with additional nutritional preparations (protein powder, multivitamin-mineral complex, vitamin D, calcium preparations)				x^2=4.040	0.094
No	1（50.00）	308（64.00）	266（58.00）		
Yes	1（50.00）	173（36.00）	193（42.00）		
Exercise frequency				x^2=25.004	＜0.001
Hardly exercise	1（50.00）	100（20.79）	60（13.10）		
1-2 times/week	1（50.00）	20（4.16）	10（2.20）		
3-5 times/week	0（0.00）	22（4.57）	17（3.70）		
6-7 times/week	0（0.00）	339（70.48）	372（81.00）		
Exercise time per exercise (Minutes)				x^2=9.809	0.019
0-29	2（100.00）	120（24.90）	93（20.30）		
30-59	0（0.00）	152（31.60）	176（38.30）		
≥60	0（0.00）	209（43.50）	190（41.40）		
Do you smoke				x^2=6.276	0.034
No	1（50.00）	432（89.80）	426（92.80）		
Yes	1（50.00）	49（10.20）	33（7.20）		
Do you consume alcohol				x^2=1.368	0.542
No	2（100.00）	386（80.20）	381（83.00）		
Yes	0（0.00）	95（19.80）	78（17.00）		
Whether you have chronic diseases				x^2=4.325	0.092
No	2（100.00）	145（30.10）	150（32.70）		
Yes	0（0.00）	336（69.90）	309（67.30）		
Number of takeaway or restaurant meals selected in a week				5.372	0.980

<1 time	2（100.00）	447（92.93）	424（92.40）		
1-2 times	0（0.00）	25（5.20）	25（5.40）		
3-4 times	0（0.00）	3（0.62）	3（0.70）		
5 times and more	0（0.00）	6（1.25）	7（1.50）		
Meal Regularity in the Last 3 Months				x^2=6.082	0.548
Irregular	0（0.00）	3（0.60）	2（0.40）		
general	0（0.00）	10（2.10）	5（1.10）		
Regular	2（100.00）	468（97.30）	452（98.50）		
Self-assessed health status in the last 3 months				x^2=13.093	0.009
Poor	0（0.00）	25（5.20）	27（5.90）		
Moderate	0（0.00）	40（8.30）	71（15.50）		
Good	2（100.00）	416（86.50）	361（78.60）		
Self-assessed mental status in the last 3 months				x^2=20.471	0.001
Poor	0（0.00）	5（1.00）	11（2.40）		
Moderate	0（0.00）	15（3.10）	42（9.20）		
Good	2（100.00）	461（95.80）	406（88.50）		
Self-assessment of Self-care Skills in Daily Life				x^2=12.048	0.059
Poor	0（0.00）	0（0.00）	1（0.20）		
Moderate	0（0.00）	12（2.50）	3（0.70）		
Good	2（100.00）	469（97.50）	455（99.10）		
Whether you can reach the nearest supermarket/grocery store in your neighborhood by walking for 15min				x^2=1.063	1
No	0（0.00）	16（3.30）	16（3.50）		
Yes	2（100.00）	465（96.70）	443（96.50）		
Whether you can reach the nearest fitness facility/gym in your neighborhood within 15min on foot				x^2=8.909	0.012
No	0（0.00）	22（4.60）	43（9.40）		
Yes	2（100.00）	459（95.40）	416（90.60）		
Whether you can reach the nearest healthcare facility by walking for 15 min				x^2=11.165	0.005
No	2（100.00）	32（6.70）	37（8.10）		
Yes	0（0.00）	449（93.30）	422（91.90）		
Whether your community provides free canteens for you				x^2=3.039	0.200
No or unknown	1（50.00）	417（86.70）	404（88.00）		
Yes	1（50.00）	64（13.30）	55（12.00）		
Whether you have used the free medical examination services provided by the community hospital in the last year				x^2=7.915	0.019
No	0（0.00）	45（9.40）	22（4.80）		
Yes	2（100.00）	436（90.60）	437（95.20）		
Level of awareness of nutrition policies				x^2=61.265	＜0.001
Unfamiliar	1（50.00）	465（96.70）	381（83.00）		
Moderate	1（50.00）	10（2.10）	28（6.10）		
Familiar	0（0.00）	6（1.20）	50（10.90）		
SARC-CalF Classification				x^2=3.335	0.199
Non-sarcopenia	2（100.00）	421（87.50）	418（91.10）		
Sarcopenia	0（0.00）	60（12.50）	41（8.90）		
Self-efficacy	33.50±6.36	29.00±5.59	32.69±4.96	F=57.225	＜0.001
Social support	38.50±0.71	43.91±6.76	38.63±7.30	F=69.572	0.001

Table 2 Awareness Level Grading and Binary Logistic Analysis of sarcopenia

	β	SE	Wald x^2	*P*	OR	95%CI
Self-efficacy	0.173	0.017	99.08	＜0.001	1.189	(0.139, 0.207)
Social support	-0.12	0.014	71.497	＜0.001	0.887	(-0.148, -0.093)
Religious belief or not（ref: Yes）						
No	-1.79	0.248	52.258	＜0.001	0.167	(-2.275, -1.305)
Educational Level（ref: College and Bachelor's degree or above）						
Primary School and Below	-0.911	0.371	6.02	0.014	0.402	(-1.639, -0.183)
Junior High School	-0.384	0.368	1.089	0.297	0.681	(-1.105, 0.337)
High School/Junior College	-0.874	0.393	4.939	0.026	0.417	(-1.645, -0.103)
Occupation type（ref: Other）						
Government employee, public sector worker	1.235	0.622	3.942	0.047	3.438	(0.016, 2.454)
Corporate/ Company personnel	0.772	0.608	1.61	0.204	2.164	(-0.420, 1.965)
Service worker	1.944	0.744	6.823	0.009	6.987	(0.485, 3.402)
Farmer	0.37	0.596	0.386	0.535	1.448	(-0.798, 1.539)
Laborer	0.832	0.613	1.843	0.175	2.298	(-0.369, 2.032)
Self-employed individual	1.117	0.736	2.302	0.129	3.056	(-0.326, 2.561)
Type of medical insurance（ref: Other）						
Basic medical insurance for urban worker	-1.615	0.846	3.648	0.056	0.199	(-3.273, 0.042)
Basic medical insurance for urban and rural resident	-1.153	0.832	1.918	0.166	0.316	(-2.784, 0.479)
Public medical insurance	-1.061	1.004	1.118	0.290	0.346	(-3.028, 0.906)
The proportion of daily high-quality protein intake to the recommended dietary intake（ref: ≥100%）						
＜75%	-13.504	0.199	4585.786	＜0.001	1.365	(-13.895, -13.113)
75%-100%	-13.184	0	.	.	1.880	(-13.184, -13.184)
Exercise frequency（ref: 6-7 times/week）						
Hardly exercise	-1.288	0.407	10.02	0.002	0.276	(-2.085, -0.49)
1-2 times/week	-0.335	0.502	0.445	0.505	0.715	(-1.318, 0.649)
3-5 times/week	0.222	0.431	0.265	0.607	1.249	(-0.623, 1.066)
Exercise time per exercise（ref: ≥60 minutes）						
1-29	0.366	0.374	0.957	0.328	1.442	(-0.367, 1.099)
30-59	-0.046	0.197	0.055	0.815	0.955	(-0.431, 0.339)
Do you smoke（ref: Yes）						
No	0.69	0.297	5.404	0.020	1.994	(0.108, 1.272)
Self-assessed health status in the last 3 months（ref: Good）						
Poor	-0.682	0.408	2.79	0.095	0.506	(-1.482, 0.118)
Moderate	0.411	0.288	2.041	0.153	1.508	(-0.153, 0.975)

Self-assessed mental status in the last 3 months（ref: Good）						
Poor	0.727	0.677	1.153	0.283	2.069	(-0.600, 2.055)
Moderate	1.092	0.4	7.442	0.006	2.980	(0.307, 1.877)
Whether you can reach the nearest fitness facility/gym in your neighborhood within 15min on foot（ref: Yes）						
No	0.709	0.342	4.309	0.038	2.032	(0.040, 1.379)
Whether you can reach the nearest healthcare facility by walking for 15 min（ref: Yes）						
No	-0.138	0.339	0.167	0.683	0.871	(-0.804, 0.527)
Whether you have used the free medical examination services provided by the community hospital in the last year（ref: Yes）						
No	-0.897	0.359	6.247	0.012	0.408	(-1.601, -0.194)
Level of awareness of nutrition policies（ref: Familiar）						
Unfamiliar	-2.25	0.505	19.858	＜0.001	0.105	(-3.240, -1.260)
Moderate	-0.832	0.643	1.671	0.196	0.435	(-2.093, 0.429)
